# Psychometric properties and measurement equivalence of the Multidimensional Fatigue Syndrome Inventory- Short Form (MFSI-SF) amongst breast cancer and lymphoma patients in Singapore

**DOI:** 10.1186/s12955-018-0846-6

**Published:** 2018-01-19

**Authors:** Alexandre Chan, Claire Lew, Xiao Jun Wang, Terence Ng, Jung-woo Chae, Hui Ling Yeo, Maung Shwe, Yan Xiang Gan

**Affiliations:** 1 Department of Pharmacy, National University of Singapore, Blk S4A Level 3, 18 Science Drive 4, Singapore, 117543 Singapore; 2 Department of Pharmacy, National Cancer Centre Singapore, Singapore, Singapore; 3 Duke-NUS Graduate Medical School Singapore, Singapore, Singapore; 4 Department of Medical Oncology, National Cancer Centre Singapore, Singapore, Singapore; 5 College of Pharmacy, Chungnam National University, Daejeon, South Korea

**Keywords:** Psychometrics, Cancer, Fatigue, Health-related quality of life, Outcome assessment (health care)

## Abstract

**Background:**

Currently, several fatigue measurement instruments are available to evaluate and measure cancer-related fatigue. Amongst them, Multidimensional Fatigue Syndrome Inventory-Short Form (MFSI-SF) is a self-reported instrument and a multidimensional scale that aims to capture the global, somatic, affective, cognitive and behavioural symptoms of fatigue. This study examines the psychometric properties and measurement equivalence of the English and Chinese versions of MFSI-SF in breast cancer and lymphoma patients in Singapore.

**Methods:**

Patients were recruited from National Cancer Centre Singapore. Validity, reliability and responsiveness of MFSI-SF were evaluated in this study. Convergent validity was evaluated by correlating total and subscales of MFSI-SF to known related constructs in EORTC QLQ-C30. Known group validity was assessed based on patients’ cancer stage, pain, insomnia and depression symptoms. Reliability was evaluated by Cronbach’s α. Responsiveness analyses were performed with patients who have undergone at least one cycle of chemotherapy. Multiple regression was used to compare the total and subscale scores of MSFI-SF between the two language versions.

**Results:**

Data from 246 (160 English and 86 Chinese version) breast cancer and lymphoma patients were included in the study. Moderate to high correlations were observed between correlated MFSI-SF subscales and EORTC QLQ-C30 domains (|r| = 0.524 to 0.774) except for a poor correlation (*r* = 0.394) observed between MFSI-SF vigour subscale and EORTC QLQ-C30 role functioning subscale. Total MFSI-SF scores could differentiate between patients with higher depression, pain and insomnia status. Internal consistency of MFSI-SF was also high (α = 0.749 to 0.944). Moderate correlation was observed between change in total MFSI-SF score and change in fatigue symptom scale score and global QoL score on EORTC QLQ-C30 (|r| = 0.478 and 0.404 respectively). Poor correlations were observed between change in scores of hypothesised subscales (|r| = 0.202 to 0.361) except for a moderate correlation between change in MFSI-SF emotional fatigue score and change in EORTC QLQ-C30 emotional functioning domain score. Measurement equivalence was established for all subscales and total MFSI-SF score except for the emotional and vigour subscales.

**Conclusions:**

This study supports the use of MFSI-SF as a reasonably valid scale with good internal consistency for measuring fatigue levels in the Singapore cancer population.

**Electronic supplementary material:**

The online version of this article (10.1186/s12955-018-0846-6) contains supplementary material, which is available to authorized users.

## Background

Cancer-related fatigue (CRF) has been reported as one of the most distressing symptoms of cancer and cancer treatments, and the prevalence ranges from 25% to 99% depending on different patient demographics, types of cancer and treatment received [1]. CRF has also been shown to be more severe and debilitating than normal fatigue experienced by people without cancer [2, 3]. However, CRF is often undermined and underdiagnosed by caregivers and clinicians due to the subjective nature of fatigue and the lack of diagnostic framework. Hence, there is a need for well-validated instruments to measure fatigue in order to facilitate diagnosis and management.

Currently, several fatigue measurement instruments are available to evaluate and measure CRF [1]. Amongst them, Multidimensional Fatigue Syndrome Inventory-Short Form (MFSI-SF) is a self-reported instrument and a multidimensional scale that aims to capture the global, somatic, affective, cognitive and behavioural symptoms of fatigue [4]. The psychometric properties of the English version of MFSI-SF was previously validated in the United States, and its factor structure was confirmed in majority breast cancer patients [5]. A Chinese version of the MFSI-SF has also been examined for its psychometric properties in Taiwan [6]. However, cross cultural adaptations of Health-Related Quality of Life instruments could possibly affect the validity of a questionnaire [7]. Given Singapore’s unique multi-ethnic demographic, it is important to evaluate the psychometric properties of the English and Chinese versions of the MFSI-SF within the Asian cancer population in Singapore.

To ensure that MFSI-SF is a valid and reliable scale for use in the Singapore cancer population, the primary objective of this study is to evaluate the psychometric properties (validity, reliability and responsiveness) of both the English and Chinese versions of the MFSI-SF. Furthermore, we also aim to determine the measurement equivalence between the two language versions.

## Methods

### Subjects and study design

This was a single-center, prospective study conducted at the outpatient clinics of the National Cancer Centre Singapore (NCCS) between 2014 and 2017. In this study, patients recruited 1) had a diagnosis of breast cancer or lymphoma 2) were at least 21 years old, 3) on or scheduled to receive chemotherapy, 4) ambulatory in nature [defined as an Eastern Cooperative Oncology Group (ECOG) performance status^1^ score of 0 or 1], 5) able to speak English or Chinese, and 6) were willing to give their informed consent [8]. The patients were excluded from the study if they were at the last time point of chemotherapy, have breast cancer or lymphoma as a secondary metastasis and physically or mentally incapable of giving written consent. This study was approved by SingHealth Institutional Ethics Review Board before commencement.

### Study procedure

Patients were requested to self-administer either the English or Chinese version of two questionnaires – MFSI-SF and the European Organisation for Research and Treatment of Cancer Quality of Life Core Questionnaire 30 (EORTC-QLQ-C30) in a 30-min period (T1). Choice of the version of the questionnaire to be administered was based on patient’s language preference and proficiency. Subsequently, the same version of the questionnaire was administered to the patients at their next follow up appointment (T2). At the time of follow-up, patients must have undergone at least one round of chemotherapy after T1.

### Psychometric assessments of CRF

#### MFSI-SF

The MFSI-SF is a 30-item multidimensional tool used to measure CRF experienced in the past week [5]. It consists of 5 empirically derived subscales: general, physical, emotional, mental and vigour. For each item, patients indicated their response on a 5-point Likert scale from 0 (“not at all”) to 4 (“extremely”). Scores on the individual subscales were then tabulated by summing the item scores for each subscale. Each subscale score ranges from 0 to 24. The Total MSFI-SF score was tabulated by adding the general, physical, emotional and mental subscale scores and subtracting vigour subscale score [5]. Total MFSI-SF score ranges from − 24 to 96 with a higher score indicating higher levels of CRF experienced by the patient. The translation process closely followed the guidelines stipulated by the Translation and Cultural Adaptation-Principles of Good practice [9]. The English version of MFSI-SF was first translated to the Chinese version by two co-investigators (within the research group) who are proficient in both languages, followed by backward- translation by two other trained bilingual investigators independently. Any discrepancies with translation were resolved through discussion. After proof reading of the final questionnaire, a pilot study was conducted in a small group of cancer patients to test for understanding. The questionnaire was then further revised based on patients’ feedback to obtain the final version used in this study.

#### EORTC QLQ-C30

The EORTC QLQ-C30 is a cancer specific tool to assess patients’ health related quality of life (HRQoL) in the past week [10]. The questionnaire consists of 30 items that includes a global QoL scale, five functional scales (physical, role, emotional, cognitive and social); three symptom scales (fatigue, nausea/vomiting and pain); six single items (dyspnea, insomnia, appetite loss, constipation, diarrhoea and financial stability). All EORTC QLQ-C30 items are rated on a four-point likert scale (‘not at all = 1′, ‘a little = 2′, ‘quite a bit = 3′ and ‘very much = 4′) except the two items assessing global QoL that uses a seven-point scale. Raw scores were linearly transformed to a 0 to 100 scale, where a higher score represent better functioning, better QoL or worse symptoms respectively. For this study, the domains in EORTC QLQ-C30 were used to establish the validity of MFSI-SF as both the English and Chinese version of the EORTC QLQ-C30 were previously validated in Singapore [11–13].

### Statistical analysis

SPSS statistics version 24 was used for all statistical analysis. Missing values from EORTC QLQ-C30 were managed as stipulated in the scoring manual. Patients with incomplete responses from MFSI-SF were excluded from analysis. Descriptive statistics were used for demographics and clinical characteristics of the patients. Baseline characteristics of the English and Chinese population were also compared. Independent samples t-test was used for continuous data that was normally distributed, whereas Mann-Whitney U test was used for data that was not normally distributed. Categorical data were compared using Chi-square tests. Significance tests were conducted at a significance level of 0.05 (two-tailed). To examine the psychometric properties, the validity, reliability and responsiveness of MFSI-SF were evaluated. Data collected from patients at T1 were used to examine the validity, reliability and measurement equivalence of the MFSI-SF. Patients who had data at both T1 and T2 were included for responsiveness analyses.

#### Validity

To assess whether MFSI-SF is a valid measure for patient’s fatigue level, a correlation analysis with EORTC QLQ-C30 was done. EORTC QLQ-C30 was chosen as the reference for validation as it has been well-validated in both the local and international population [10–12, 14–18]. Spearman correlation was used for all correlation analysis as data obtained cannot be approximated to a normal distribution. A statistically significant correlation was indicated by a *P* <  0.05. Correlation values of < 0.4 are poor; 0.4–0.7 are moderate and > 0.7 are strong correlations [19].

To evaluate the construct validity of MFSI-SF, we compared total and subscale MFSI-SF scores to their known related construct in EORTC QLQ-C30. As studies have shown that CRF usually coexists with symptoms of pain, insomnia, and dyspnoea [20–22], we hypothesised that total MFSI-SF score would have a moderate to strong (|r| ≥ 0.4) correlation with the pain symptom subscale as well as the insomnia and dyspnoea items on EORTC QLQ-C30. Moreover, CRF is closely associated with patients’ QoL [23, 24]. Hence, we hypothesised that the global QoL scale on the EORTC QLQ-C30 would correlate with total MFSI-SF score. For the subscales of MFSI-SF, we hypothesised that the physical, emotional, mental and vigour subscale on MFSI-SF would correlate with the physical, emotional, cognitive and role functioning subscales on EORTC QLQ-C30. Lastly, total and subscale scores of MFSI-SF would correlate with the fatigue symptom scale on EORTC QLQ-C30. All hypothesised correlations were expected to be moderate to strong (|r| ≥ 0.4).

Divergent validity was assessed to evaluate whether scales in EORTC QLQ-C30 not associated with CRF would be poorly correlated with MFSI-SF scores. We hypothesised that total and subscale MFSI-SF score would be poorly correlated to constipation and diarrhoea items on EORTC QLQ_C30 (|r| <  0.4).

Known group validity was performed to assess MFSI-SF’s ability to differentiate between differing groups known to have characteristics that affect fatigue levels. Studies have shown that CRF is associated with more advanced cancer staging [24, 25]. Hence, we hypothesised that patients with more advanced cancer stage (I-II vs. III-IV) would have a higher MFSI-SF total score. Furthermore, CRF is associated with depression, insomnia and pain [20–22, 25–27]. Hence, we hypothesised that patients with a higher score on EORTC QLQ-C30 item 24 “Did you feel depressed”, item 11 “Have you had trouble sleeping” and item 9 “Have you had pain” (1–2 vs. 3–4) would have a higher MFSI-SF score. Based on the established Minimal Clinically Important Difference (MCID) of MFSI-SF (4.50–10.79 points), we hypothesized that a score difference of ≥10 points between the groups represent a clinically significant difference [28]. To evaluate the statistical significance, Mann-Whitney U tests were used to compare the scores between the known groups.

#### Reliability

Internal consistency of MFSI-SF was evaluated using Cronbach’s alpha coefficient (α). An α value of above 0.7 was regarded as having satisfactory consistency [29]. An item-to-subscale correlation analysis was also performed to identify problematic or inconsistent items in the respective MFSI-SF subscale. Correction overlap was applied by calculating the corrected item-to-subscale correlation for each item after removing its contribution to subscale scores. Any items with a correlated item-to-scale correlation of more than 0.3 were considered acceptable.

#### Responsiveness

Responsiveness refers to the ability of a scale to detect changes over time in the construct to be measured [30]. In this case, responsiveness of MFSI-SF refers to the ability of MFSI-SF to detect changes in patient’s fatigue level following known interventions. Patients were followed-up after they have undergone at least one or more chemotherapy (T2) as studies showed that patient’s fatigue level changes with consecutive cycles of chemotherapy [31, 32]. The change in EORTC QLQ-C30 domain scores was used as the reference to evaluate the responsiveness of MFSI-SF as the responsiveness of EORTC QLQ-C30 was demonstrated in previous studies [17, 33].

Treating responsiveness as a longitudinal aspect of validity, we used the criterion approach to evaluate responsiveness. We compared the change in total and subscales MFSI-SF scores to the change in scores of known related constructs in EORTC QLQ-C30. We hypothesised that an improvement in total MFSI-SF score (i.e. a lower score) would correspond to lower scores on the fatigue, pain, dyspnoea and insomnia symptom scale on the EORTC QLQ-C30. We also hypothesised that an improvement in total MFSI-SF score would correspond to an improvement in global QoL score on the EORTC QLQ-C30.

For the change in subscales score of MFSI-SF, we hypothesised that an improvement in physical, emotional, mental and vigour subscales score on MFSI-SF would correspond to an improvement in physical, emotional, cognitive and role functioning score on EORTC QLQ-C30 respectively. In addition, improvements in total and subscale scores of MFSI-SF were hypothesised to correlate with improvements in the fatigue symptom scale on EORTC QLQ-C30. All related correlations were hypothesised to be moderate to strong (|r| ≥ 0.4).

In addition to establishing the longitudinal validity (responsiveness) using the construct approach, the ability of MFSI-SF to detect clinically significant changes in scores was also evaluated. Based on the MCID established, a score difference of ≥10 between T1 and T2 was regarded as a clinically significant change [28].

#### Measurement equivalence

Measurement equivalence evaluates the similarity in psychometric properties between the English and Chinese version. Using the methodology for assessing therapeutic equivalence in clinical trials, the 95% CIs of MFSI-SF total and subscales score differences were compared with predefined equivalence margins to determine whether differences in scores were clinically important [34, 35]. As score differences between the Chinese and English versions could be due to underlying differences in baseline characteristics, a univariate analysis was first performed to single out variables that were statistically significant between patients completing the Chinese and English version of the MFSI-SF. Subsequently, multiple regression analysis was performed to tabulate the 95% CIs of the score differences after adjusting for these variables. Equivalence was established if the 95% CIs of the adjusted mean differences fell within predefined equivalence margin of ±0.5 [35, 36].

## Results

### Demographic characteristics

255 patients were recruited in the study. Out of 255 patients, 8 patients (3.1%) had incomplete responses to MFSI-SF and were excluded from the study, 1 patient (0.4%) withdrew from the study.

A total of 246 breast cancer and lymphoma patients were included for analysis. One hundred and sixty patients (65.0%) completed the English version of the MFSI-SF while 86 patients (35.0%) completed the Chinese version of MFSI-SF (Table 1). There were 6 missing responses (0.08%) for 6 different items on the EORTC QLQ-C30 and they were managed as stipulated in the scoring manual. The six missing responses were from EORTC QLQ-C30 items 1, 2, 3, 11, 17, 24. Majority of the patients were females (93.5%), Chinese (80.5%), breast cancer patients (91.5%), married (73.6%) and have an ECOG performance status of 0 (89.8%). Statistically significant differences were observed for age, race, education levels, ECOG status and menopausal status between patients who completed the English and Chinese version of the MFSI-SF.

**Table 1:** Baseline demographic and clinical information of patients

	Total (*N* = 246)	English Version (*n* = 160)	Chinese Version (*n* = 86)	*P*-value
Demographic information				
Age, mean (± SD)	53.4 (±10.0)	51.2 (± 10.3)	57.4 (±8.0)	< 0.001^#^
Gender, n (%)				0.192
Female	230 (93.5)	152 (95.0)	78 (90.7)	
Male	16 (6.5)	8 (5.0)	8 (9.3)	
Race, n (%)				< 0.001^#^
Chinese	198 (80.5)	114 (71.3)	84 (97.7)	
Non-Chinese				
Malay	26 (10.6)	26 (16.3)	0 (0.0)	
Indians	12 (4.9)	12 (7.5)	0 (0.0)	
Others	10 (4.1)	8 (5.0)	2 (2.3)	
Marital Status, n (%)				
Single	45 (18.3)	32 (20.0)	13 (15.1)	0.546
Married	181 (73.6)	116 (72.5)	65 (75.6)	
Divorced	17 (6.9)	11 (6.9)	6 (7.0)	
Widowed	3 (1.2)	1 (0.6)	2 (2.3)	
Education, n (%)				< 0.001^#^
Low Education	42 (17.1)	6 (3.8)	36 (41.9)	
None	9 (3.7)	2 (1.3)	7 (8.1)	
Primary	33 (13.4)	4 (2.5)	29 (33.7)	
High Education	204 (82.9)	154 (96.3)	50 (58.1)	
Secondary	111 (45.1)	73 (45.6)	38 (44.2)	
Pre-University	45 (18.3)	37 (23.1)	8 (9.3)	
Graduate and Above	48 (19.5)	44 (27.5)	4 (4.7)	
Occupation, n (%)				0.097
Employed	130 (52.8)	93 (58.1)	37 (43.0)	
Unemployed	90 (36.6)	53 (33.1)	37 (43.0)	
Student	1 (0.4)	1 (0.6)	0 (0.0)	
Retired	25 (10.2)	13 (8.1)	12 (14.0)	
Clinical Information				
ECOG, n (%)				0.030^#^
0	221 (89.8)	149 (93.1)	72 (83.7)	
1	15 (6.1)	6 (3.8)	9 (10.5)	
Missing	10 (4.1)	5 (3.1)	5 (5.8)	
Menopausal status, n (%)				< 0.001^#^
Pre-menopausal	98 (39.8)	79 (49.4)	19 (22.1)	
Post-menopausal	129 (52.4)	71 (44.4)	58 (67.4)	
Not Applicable	16 (6.5)	8 (5.0)	8 (9.3)	
Missing	3 (1.2)	2 (1.3)		
Cancer Diagnosis, n (%)				0.427
Breast	225 (91.5)	148 (92.5)	77 (89.5)	
Lymphoma	21 (8.5)	12 (7.5)	9 (10.5)	
Cancer Stage, n (%)				0.554
I	34 (13.8)	26 (16.3)	8 (9.3)	
II	134 (54.5)	87 (54.4)	47 (54.7)	
III	50 (20.3)	31 (19.4)	19 (22.1)	
IV	23 (9.3)	15 (9.4)	8 (9.3)	
Unknown	5 (2.0)	1 (0.6)	4 (4.7)	
Chemotherapy Regimen, n (%)				0.819
Anthracycline containing	132 (53.7)	85 (53.1)	47 (54.7)	
Non-anthracycline containing	114 (46.3)	75 (46.9)	39 (45.3)	

*ECOG* Eastern Cooperative Oncology Group, *Pre-M* pre-menopausal, *Post-M* post-menopausal

^#^Denotes statistically significant difference (*P* < 0.05)

### Validity

Correlation coefficients can be found in Table 2 and Additional file 1: Table S1. All hypothesised correlation coefficients performed in the expected direction and magnitude except for the correlation between the vigour subscale and role functioning subscale, total MFSI-SF score and EORTC QLQ-C30 pain symptom score and insomnia item score. Based on the criteria set for correlation coefficients interpretation, a moderate correlation was observed between total MFSI-SF score and EORTC QLQ-C30 global QoL scale, fatigue symptom scale and dyspnoea item scores (|r| = 0.419 to 0.667). A poor correlation was observed between total MFSI-SF score and EORTC QLQ-C30 pain symptom scale and insomnia symptom item scores (|r| = 0.378 and 0.386 respectively). Correlating MFSI-SF’s subscales, a high correlation was observed between the emotional and mental fatigue subscales of the MFSI-SF and the emotional and cognitive functioning domains on the EORTC QLQ-C30 (*r* = − 0.774 and *r* = − 0.756, respectively). The physical subscale had a moderate correlation with the EORTC QLQ-C30 physical functioning domains (*r* = − 0.524) and a poor correlation was observed between the vigour subscale and the EORTC QLQ-C30 role functioning domain (*r* = 0.394). As expected, total and subscale scores of MFSI-SF were moderately correlated (|r| = 0.451 to 0.655) to EORTC QLQ-C30 fatigue symptom scale.

**Table 2:** Spearman’s correlations between MFSI-SF subscales and EORTC QLQ-C30 domains

EORTC QLQ-C30	MFSI-SF					
	General Fatigue	Physical Fatigue	Emotional Fatigue	Mental Fatigue	Vigour	Total Score
Functional						
Physical	− 0.501^a^	− **0.524**^a^	− 0.404^a^	− 0.347^a^	0.358^a^	− 0.515^a^
Role	− 0.507^a^	− 0.397^a^	− 0.412^a^	− 0.390^a^	**0.394** ^a^	− 0.501^a^
Emotional	− 0.581^a^	− 0.492^a^	− **0.774**^a^	− 0.482^a^	0.488^a^	− 0.661^a^
Cognitive	− 0.416^a^	− 0.383^a^	− 0.476^a^	**− 0.756** ^a^	0.324^a^	− 0.519^a^
Social	− 0.509^a^	− 0.421^a^	− 0.485^a^	− 0.500^a^	0.478^a^	− 0.591^a^
Global QOL						
Global health status (GHS)	− 0.559^a^	− 0.533^a^	− 0.492^a^	− 0.436^a^	0.612^a^	− **0.667**^a^
Symptom scales						
Fatigue	**0.655** ^a^	**0.537** ^a^	**0.517** ^a^	**0.451** ^a^	− **0.463**^a^	**0.627** ^a^
Pain	0.374^a^	0.461^a^	0.367^a^	0.244^a^	− 0.230^a^	**0.378** ^a^
Symptom Items						
Dyspnoea	0.410^a^	0.365^a^	0.363^a^	0.366^a^	− 0.287^a^	**0.419** ^a^
Insomnia	0.367^a^	0.398^a^	0.360^a^	0.319^a^	− 0.234^a^	**0.386** ^a^
Appetite loss	0.365^a^	0.378^a^	0.323^a^	0.232^a^	− 0.380^a^	0.410^a^
Constipation	**0.348** ^a^	**0.265** ^a^	**0.225** ^a^	**0.206** ^a^	− **0.267**^a^	**0.323** ^a^
Nausea & vomiting	0.254^a^	0.229^a^	0.218^a^	0.127^b^	− 0.221^a^	0.272^a^
Diarrhoea	**0.124**	**0.248** ^a^	**0.113**	**0.211** ^a^	− **0.089**	**0.176** ^a^
Financial Difficulties	0.392	0.364^a^	0.416^a^	0.324^a^	− 0.378^a^	0.470^a^

*MFSI-SF* Multidimensional Fatigue Symptom Inventory - Short Form, *EORTC QLQ-C30* European Organization for Research and Treatment of Cancer Quality of Life Core Questionnaire 30

Unmarked correlations were not significant at the 0.05 level

^a^Correlation was significant at the 0.01 level (2-tailed)

^b^Correlation was significant at the 0.05 level (2 tailed)

Bolded values indicate hypothesised correlations

Further subgroup analysis was done based on the two language versions (Additional file 1: Table S1). Results obtained were largely similar as when results are pooled. However, a moderate correlation (*r* = 0.451) was obtained between MFSI-SF vigour subscale and EORTC QLQ C-30 role functioning domain for the English version while a poor correlation (*r* = 0.316) was obtained for the Chinese version. Likewise, a moderate correlation (*r* = 0.440) was obtained between total MFSI-SF score and EORTC QLQ-C30 dyspnoea items score for the English version while a poor correlation (*r* = 0.386) was obtained for the Chinese version. In addition, a poor correlation (*r* = 0.390) was observed between MFSI-SF mental subscale and EORTC QLQ-C30 fatigue symptom scale for the English version.

With regards to divergent validity, the total and subscale scores of the MFSI-SF were poorly correlated (|r| = 0.034 to 0.348) to the constipation and diarrhoea items as expected. Similar results were obtained in the subgroup analysis based on MFSI-SF language version (Table 2 and Additional file 1: Table S1).

Raw mean scores showed that total and subscale MFSI-SF scores were in line with hypotheses for the known groups (Table 3). Higher MFSI-SF scores were obtained for those with more advanced cancer staging and higher pain, depression and insomnia status.

**Table 3:** Known group validity of MFSI-SF according to ECOG performance status, cancer staging, co-morbidity, pain and insomnia status

Subscales Median scores (IQR)	Breast cancer stage^a^			EORTC “Did you feel depressed” item 24 score			EORTC “Have you had trouble sleeping” item 11 score^b^			EORTC “Have you had pain” item 9 score		
	1–2 (*n* = 168)	3–4 (*n* = 73)	Score difference	1–2 (*n* = 232)	3–4 (*n* = 13)	Score difference	1–2 (*n* = 189)	3–4 (*n* = 56)	Score difference	1–2 (*n* = 220)	3–4 (*n* = 26)	Score difference
General	3.00 (1.00, 5.00)	5.00 (1.00, 9.00)	2.00^#^	3.00 (1.00, 6.00)	10.00 (8.50, 16.50)	7.00^#^	2.00 (0.50, 5.00)	6.00 (3.00, 11.75)	4.00^#^	3.00 (1.00, 5.75)	9.00 (4.00, 14.25)	6.00^#^
Physical	2.00 (1.00, 5.75)	4.00 (1.50, 6.50)	2.00^#^	2.00 (1.00, 5.75)	9.00 (6.00, 13.00)	7.00^#^	2.00 (0.00, 4.50)	6.00 (3.00, 9.00)	4.00^#^	2.00 (1.00, 5.00)	8.50 (4.00, 15.00)	6.50^#^
Emotional	1.00 (0.00, 5.00)	2.00 (0.00, 5.00)	1.00	1.00 (0.00, 5.00)	12.00 (9.00, 13.00)	11.00^#^	1.00 (0.00, 5.00)	4.00 (1.00, 8.75)	3.00^#^	1.00 (0.00, 5.00)	6.50 (2.00, 10.50)	5.50^#^
Mental	1.00 (0.00, 5.00)	2.00 (0.00, 5.50)	1.00	1.50 (0.00, 4.00)	10.00 (5.50, 13.50)	8.50^#^	1.00 (0.00, 4.00)	2.50 (0.25, 7.75)	1.50^#^	1.50 (0.00, 4.00)	5.00 (0.75, 9.25)	3.50^#^
Vigour	14.00 (8.00, 19.00)	14.00 (10.00, 18.50)	0.00	14.00 (9.25 19.00)	4.00 (1.50, 8.50)	− 10.00^#^	15.00 (10.00, 19.00)	12.00 (6.00, 16.50)	− 3.00^#^	14.00 (9.00, 19.00)	11.00 (6.00, 15.25)	− 3.00^#^
Total MFSI-SF score	− 3.00 (− 15.00, 9.00)	− 1.00 (− 11.00, 13.00)	2.00	− 3.00 (− 15.00, 8.75)	35.00 (30.00, 54.50)	38.00^#^	− 5.00 (− 15.00, 7.00)	9.50 (− 5.75, 34.75)	14.50^#^	− 4.00 (−15.00, 8.00)	17.00 (1.00, 36.75)	21.00^#^

*MFSI-SF* Multidimensional Fatigue Symptom Inventory - Short Form, *EORTC QLQ-C30* European Organization for Research and Treatment of Cancer Quality of Life Core Questionnaire 30

^#^Denotes statistically significance difference (P < 0.05)

^a^Data missing for 5 patients

^b^Data missing for 1 patient

Clinically significant differences were observed in total MFSI-SF scores (score difference ≥ 10 points) of patients with different depression, insomnia and pain status. These differences were also statistically significant (all *p* <  0.05). Amongst patients with different cancer staging, the general and physical fatigue subscales showed statistically significant differences in scores.

### Reliability

Results showed satisfactory internal consistency (α = 0.749 to 0.944) and acceptable item-to-subscale correlations (*r* = 0.450 to 0.868) for both the English and Chinese versions individually and when the results were pooled for both versions (Table 4). Although all α values were satisfactory, lower internal consistency was observed across all domains for the Chinese version as compared to the English version (α =0.749 to 0.917 vs 0.889 to 0.944).

**Table 4:** Internal consistency of the MFSI-SF subscales and the corrected item-to-domain Spearman’s correlations

Domain/Scale	Item	Content (Listed in English)	Total MFSI-SF score of English and Chinese versions (N = 246)		English version of MFSI-SF (n = 160)		Chinese version of MFSI-SF (n = 86)	
			Corrected item-to-domain correlation^a^	Cronbach’s α	Corrected item-to-domain correlation	Cronbach’s α	Corrected item-to-domain correlation	Cronbach’s α
General (6 items)				0.914		0.917		0.904
	10	I feel pooped	0.611		0.530		0.765	
	12	I feel worn out	0.726		0.694		0.778	
	14	I feel fatigued	0.758		0.769		0.715	
	17	I feel sluggish	0.624		0.686		0.474	
	18	I feel run down	0.732		0.761		0.747	
	28	I feel tired	0.773		0.751		0.783	
Physical (6 items)				0.863		0.889		0.749
	2	My muscles ache	0.614		0.685		0.463	
	4	My legs feel weak	0.616		0.676		0.479	
	6	My head feels heavy	0.477		0.491		0.450	
	16	My arms feel weak	0.568		0.608		0.503	
	19	I ache all over	0.632		0.702		0.476	
	26	My body feels heavy all over	0.629		0.686		0.502	
Emotional (6 items)				0.912		0.921		0.887
	3	I feel upset	0.746		0.737		0.764	
	8	I feel nervous	0.676		0.699		0.632	
	13	I feel sad	0.789		0.789		0.787	
	21	I feel depressed	0.723		0.720		0.727	
	23	I feel tense	0.685		0.732		0.600	
	30	I am distressed	0.678		0.655		0.730	
Mental (6 items)				0.921		0.928		0.889
	1	I have trouble remembering things	0.748		0.784		0.674	
	11	I am confused	0.615		0.622		0.603	
	15	I have trouble paying attention	0.729		0.780		0.635	
	20	I am unable to concentrate	0.716		0.740		0.671	
	25	I make more mistakes than usual	0.679		0.671		0.701	
	27	I am forgetful	0.781		0.798		0.756	
Vigour (6 items)				0.936		0.944		0.917
	5	I feel cheerful	0.833		0.827		0.844	
	7	I feel lively	0.820		0.868		0.719	
	9	I feel relaxed	0.790		0.839		0.690	
	22	I feel refreshed	0.833		0.829		0.843	
	24	I feel energetic	0.853		0.853		0.862	
	29	I feel calm	0.803		0.833		0.749	

All correlations were significant at the 0.01 level (two tailed)

All Cronbach’s alpha coefficient was above the satisfactory level of 0.7

All items had satisfactory item-to-domain correlation (defined by a corrected *r* > 0.300)

*MFSI-SF* Multidimensional Fatigue Symptom Inventory - Short Form

^a^The corrected item-to-domain correlation was calculated for each item by removing the contribution of the item’s score to its corresponding subscale score

### Responsiveness

Data from 224 (91.1% among total participants) patients were obtained at T2. Correlation coefficients for responsiveness can be found in Table 5. Poor correlations were observed between change in total MFSI-SF scores and change in EORTC QLQ-C30 pain symptom scale and dyspnoea and insomnia item score (|r| = 0.240 to 0.349). A moderate correlation was observed between change in total MFSI-SF score and change in EORTC QLQ-C30 fatigue symptom scale and global QoL scale (*r* = 0.478 and − 0.404 respectively) as hypothesised. For the change in MFSI-SF subscale scores, poor correlations were observed between all hypothesised correlated EORTC QLQ-C30 subscales (|r| = 0.202 to 0.361) except for a moderate correlation between change in MFSI-SF emotional fatigue subscale score and change in EORTC QLQ-C30 emotional functioning score. Change in total and subscale scores on MFSI-SF were poorly correlated (|r| = 0.005 to 0.185) with change in constipation and diarrhoea item scores as expected.

**Table 5:** Spearman’s correlations between change in scores on the MFSI-SF subscales and EORTC QLQ-C30 domains

EORTC QLQ-C30	MFSI-SF					
	General Fatigue	Physical Fatigue	Emotional Fatigue	Mental Fatigue	Vigour	Total Score
Functional						
Physical	− 0.292^a^	**− 0.239** ^a^	− 0.156^b^	− 0.173^a^	0.183^a^	− 0.297^a^
Role	− 0.262^a^	− 0.202^a^	− 0.211^a^	− 0.203^a^	**0.202** ^a^	− 0.296^a^
Emotional	− 0.236^a^	− 0.241^a^	**− 0.447** ^a^	− 0.243^a^	0.219^a^	− 0.372^a^
Cognitive	− 0.202^a^	− 0.205^a^	− 0.196^a^	− **0.361**^a^	0.183^a^	− 0.294^a^
Social	− 0.231^a^	− 0.153^a^	− 0.180^a^	− 0.124	0.156^b^	− 0.228^a^
Global QOL						
Global health status (GHS)	− 0.361^a^	− 0.286^a^	− 0.317	− 0.180^a^	0.302^a^	− **0.404**^a^
Symptom scales						
Fatigue	**0.412**	**0.368** ^a^	**0.406** ^a^	**0.277** ^a^	− **0.283**^a^	**0.478** ^a^
Pain	0.221	0.350^a^	0.113	0.127	− 0.090	**0.240** ^a^
Symptom Items						
Dyspnoea	0.170^b^	0.275^a^	0.302^a^	0.226^a^	− 0.187^a^	**0.286** ^a^
Insomnia	0.266^a^	0.309^a^	0.191^a^	0.200^a^	− 0.242^a^	**0.349** ^a^
Appetite loss	0.230^a^	0.210^a^	0.277^a^	0.194^a^	− 0.345^a^	0.354^a^
Constipation	**0.099**	**0.063**	**0.083**	**0.006**	− **0.185**^a^	**0.114**
Nausea & vomiting	0.089	0.076	0.127	0.163^b^	− 0.139^b^	0.159^b^
Diarrhoea	**0.098**	**0.153** ^b^	− **0.046**	− **0.079**	**0.005**	**0.029**
Financial Difficulties	0.132^b^	0.095	0.141	0.143^b^	− 0.267^a^	0.225^a^

*MFSI-SF* Multidimensional Fatigue Symptom Inventory - Short Form, *EORTC QLQ-C30* European Organization for Research and Treatment of Cancer Quality of Life Core Questionnaire 30

Unmarked correlations were not significant at the 0.05 level

^a^Correlation was significant at the 0.01 level (2-tailed)

^b^Correlation was significant at the 0.05 level (2-tailed)

Bolded values indicate hypothesised correlations

Based on the pre-defined clinically significant score differences (score difference ≥ 10 points), there were no clinically significant score differences observed for change in total and MFSI-SF subscale scores between T1 and T2 (Additional file 1: Table S2).

### Measurement equivalence

All statistically significant different variables between the Chinese and English populations obtained from Table 1 and Additional file 1: Table S3, except for menopausal status, were adjusted in the regression model when evaluating the measurement equivalence between the English and Chinese versions of MFSI-SF (Table 6 and Additional file 1: Table S4). Menopausal status was not included in the model as it is associated to differences in age, which was already adjusted for in the regression model. Ten patients (4.1%) with missing values for the variables adjusted were excluded from measurement equivalence analysis. Ultimately, 236 patients (95.9%) were included into the analysis. After adjusting for the covariates, the 95% CI for the adjusted difference of the general, physical, mental subscales and total MFSI-SF scores between the English and Chinese versions of MFSI-SF were within the ±0.5 S.D. margin, suggesting an acceptable measurement equivalence between the two language versions. However, the 95% CI of the adjusted difference between the English and Chinese versions for the emotional and vigour subscale exceeded the ±0.5 S.D. margin.

**Table 6:** Total and subscale score of English and Chinese MFSI-SF versions and measurement equivalence between the two versions

(Theoretical Score Range)	Mean ± S.D.			English vs. Chinese			
	Total (*N* = 236)^e^	English-version (*n* = 155)	Chinese-version (*n* = 81)	Equivalence margin^a^ (±0.25 S.D.)	Equivalence margin^b^ (±0.5 S.D.)	95% CI of Unadjusted difference	95% CI of Adjusted difference^c^
Total MFSI-SF score	0.720 (19.06)	1.56 (20.32)	− 0.889 (16.36)	± 4.59	± 9.17	− 2.450 (− 7.599 to 2.698)	0.095 (− 1.512 to 0.097)
General	4.34 (4.51)	4.87 (4.74)	3.33 (3.84)	± 1.07	± 2.15	− 1.538 (− 2.742 to − 0.333)	− 0.009 (− 1.327 to 1.148)
Physical	3.88 (4.11)	4.22 (4.51)	3.22 (3.15)	± 0.96	± 1.91	− 0.997 (− 2.103 to 0.109)	0.023 (− 0.967 to 1.357)
Emotional	3.21 (4.25)	3.34 (4.46)	2.96 (3.83)	± 1.04	± 2.07	− 0.373 (− 1.522 to 0.777)	0.096 (− 0.423 to 2.142)^d^
Mental	3.00 (3.91)	3.32 (4.28)	2.38 (3.01)	± 0.91	± 1.82	− 0.933 (− 1.985 to 0.118)	− 0.016 (− 1.274 to 1.017)
Vigour	13.70 (6.48)	13.18 (6.61)	12.79 (6.14)	± 1.59	±3.19	− 1.391 (− 3.134 to 0.353)	− 0.217 (− 4.922 to − 0.991)^d^

*MFSI-SF* Multidimensional Fatigue Symptom Inventory - Short Form

Equivalence was assessed by comparing 95% CI of the adjusted difference to the equivalence margin (±0.5 S.D.)

^a^The equivalence margin is the simple average of the standard deviation of the Chinese and English scores multiplied by 0.25

^b^The equivalence margin is the simple average of the standard deviation of the Chinese and English scores multiplied by 0.50

^c^Mean difference between the English and Chinese version was adjusted for relevant variables that showed statistical significant differences between the English and Chinese version of MFSI-SF (Table 1 and Additional file 1: Table S3): age, race (Chinese vs Non-Chinese), education (low education vs high education), ECOG status, EORTC nausea and vomiting symptom, EORTC dyspnoea item

^d^95% CI of the adjusted difference exceeded the equivalence margin threshold (±0.5 S.D.)

^e^Data for ECOG was missing for 10 patients

## Discussion

This study suggests that MFSI-SF has a reasonably satisfactory validity with good internal consistency for use in the local multi-ethnic cancer population in Singapore. This is in line with overseas validation studies of MFSI-SF which demonstrated the validity and reliability of MFSI-SF using other references for construct validity such as STAI (State-Trait Anxiety Inventory for Adults) [4, 37]. However, MFSI-SF may not be responsive to the changes in fatigue level following consecutive cycles of chemotherapy in this patient population. Measurement equivalence for the English and Chinese version was demonstrated for total MFSI-SF score based on the equivalence margin of ±0.5 S.D. However, pooled results obtained from the individual subscales of MFSI-SF should be interpreted with caution as measurement equivalences were not obtained for the emotional and vigour subscales.

For construct validity, poor correlation was observed between MFSI-SF vigour subscale and EORTC QLQ C-30 role functioning subscale. On further analyses based on language version, the poor correlation was only observed in the Chinese version of MFSI-SF. This could be attributed to poor equivalence between the English and Chinese version of the MFSI-SF vigour subscale which has also been reflected in the measurement equivalence analysis. Hence, pooled results from the vigour subscale should be interpreted cautiously and further modifications should be made to the Chinese version of MFSI-SF vigour subscale to improve equivalence.

For known group validity, higher fatigue scores were obtained for patients with more advanced cancer staging as well as more depression, insomnia and pain symptoms. However, only total MFSI-SF scores showed clinically significant differences between patients with different depression, insomnia and pain status. The inability to observe a clinically significant difference amongst patients with different cancer staging might be due to majority of patients being in stage II (54.5%) and stage III of cancer (20.3%). The difference between fatigue levels of patients in stages II and III might not be as large. Hence, clinically significant difference might not be observable when the scores are pooled with patients in stages I and IV respectively. Furthermore, as the MCID established was based on total MFSI-SF scores, it may not be as appropriate when extrapolating to clinical significance of subscale scores. This might have also contributed to the lack of clinical significance observed for most of the subscales.

MFSI-SF has also demonstrated good internal consistency in this study. This is in line with previous validation studies conducted in the United States for the English version of MFSI-SF and the Chinese version of MFSI-SF in Taiwan which obtained α > 0.7 [5, 6]. However, item 17 in the Chinese version used in this study showed a moderate item-to-domain correlation as compared to the high correlations observed for other items in the scale (*r* = 0.474). It may appear that literal equivalence is achieved with the translation, but there might be issues associated with cultural equivalence with the translation [38]. This could possibly explain the different interpretations of item 17 in the Chinese version and hence, the lower item-to-domain correlation. In the English version of MFSI-SF, a moderate correlation was observed for item 10 “I feel pooped” (*r* = 0.530) as compared to the high item-to-domain correlation for the Chinese version (*r* = 0.765). During data collection, researchers were often asked by patients to explain the meaning of “pooped” as they did not understand the term. MFSI-SF was developed in the United States and issues associated with cross cultural adaptation of the instrument might possibly explain the inability to interpret slang terms like “pooped” [39]. Furthermore, the relatively older patient population of this study (53.4 ± 10.0 years old) might further contribute to lower understanding of slang words as they might not be as exposed to foreign culture via popular media as compared to the younger population. All these could result in the lower item-to-domain correlation. As such, possible modifications to item 10 and item 17 could be done to improve the psychometric properties of the tool.

Responsiveness of a QoL tool can affect its ability to assess the effectiveness of treatment strategies or be used as a primary outcome measure to detect changes in clinical trials. Total MFSI-SF score could detect changes in patient’s fatigue levels and global QoL score. However, the ability of individual subscales of MFSI-SF to detect the magnitude of changes in relevant constructs were not as expected. The hypothesised magnitude of correlation was only observed between change in MFSI-SF emotional subscale score and change in EORTC QLQ-C30 emotional functioning score. As a group, there was also a lack of clinically significant change observed amongst patients between T1 and T2. The poor responsiveness could be partly attributed to the generally lower mean scores obtained across subscale and total MFSI-SF scores at T1 and T2 (score ranges from 0 to 24 for individual subscales and − 24 to 96 for total MFSI-SF score). During data collection, patients tend to be more conservative in choosing extreme score ranges which might have contributed to the lower mean scores obtained. Such low scores were more evident in the Chinese population (Additional file 1: Table S4). This could be reflective of a true fatigue level of our population, or this could possibly be due to certain underlying values with regards to reporting of “negative” symptoms experienced. This was evident with the EORTC QLQ-C30 scores obtained in this study, where lower scores were obtained for more subjective “negative” attributes (e.g. fatigue, pain) while “positive” attributes such as functioning had higher scores observed. The lower scores obtained might have resulted in a floor effect whereby further deterioration in fatigue scores following consecutive cycles of chemotherapy would not be clinically observable.

Measurement equivalence was not observed for the emotional and vigour subscales. This is possibly attributed to translation errors or differences in cultural interpretation for these items. Items in the emotional and vigour subscale involved asking patients on how they are feeling. Different levels of tolerance towards self-expression between the Chinese and Western culture could possibly explain for these differences, leading to a more conservative response in the Chinese version of the questionnaire as mentioned above. Hence, caution must be exercised when interpreting pooled results from these subscales.

There are several limitations to this study. Firstly, a detailed cognitive briefing of both language versions of MFSI-SF was not performed. However, the English version was heavily used and validated in numerous studies, and we had piloted the Chinese version with a few patients to ensure there is no ambiguity with the language. Secondly, factor analyses were not conducted for MFSI-SF [40]. Factor analysis is a form of construct validation and helps to explore patterns of correlations between items or for confirming the pre-existing factor structure of MFSI-SF. Factor analyses were not conducted due to limited sample size of our study. COSMIN checklist recommends a minimum subject-to-variable ratio of 4:1 for factor analyses [41]. Our sample size (*n* = 86) for the Chinese version would be inadequate for factor analyses with the 30-item MFSI-SF questionnaire [41]. However, previous validation study done in Taiwan on a Chinese version of MFSI-SF supports a four subscale structure of physical, emotional, mental and vigour subscale instead [6]. Furthermore, problematic items were also observed in the general subscale based on item-to-domain correlations analyses and discrepancies in the correlation between the MFSI-SF vigour subscale and EORTC QLQ-C30 role functioning domain was also observed following subgroup analyses based on language version. Hence, future validation studies can consider recruiting a larger sample size for Exploratory Factor Analysis (EFA) and Confirmatory Factor Analysis (CFA) on the Chinese version of MFSI-SF. Thirdly, the instrument used for construct validation is a HRQoL measure rather than a fatigue measure. Although QoL and fatigue are closely related constructs, there are still fundamental differences in their interpretation. This could potentially limit the conclusions drawn from this study. However, given that there is a lack of cancer-related fatigue questionnaires validated in the Singaporean cancer population and EORTC QLQ-C30 has been well-validated and utilised in this cancer population, EORTC QLQ-C30 was chosen as the comparator instrument for construct validation. Fourthly, responsiveness analyses were based on hypothesis testing rather than using a gold standard for comparison. As the criterion approach will provide stronger evidence for responsiveness of MFSI-SF, future studies can consider using a gold standard such as a global rating scale to evaluate changes in the fatigue levels. Lastly, MFSI-SF uses a five point Likert scale respond format. Classical test theory for evaluating psychometric properties assumes an interval or even ratio measurement between these responses [42]. However, the relative value between each option might not be equivalent. Hence, more sophisticated psychometric tests such as Rasch analysis based on Item Response Theory (IRT) could be done in future validation studies to evaluate how specific test item functions in MFSI-SF.

Ever since its development, MFSI-SF has been extensively used as a fatigue assessment tool in studies. Fatigue levels in both cancer and non-cancer patient population were assessed using MFSI-SF and demonstrated good reliability and validity [37]. Coupled with results from this study, there is a potential for the clinical utility of MFSI-SF as a fatigue assessment tool in local studies and could possibly be validated in non-cancer population in the future.

## Conclusion

MFSI-SF is a reasonably valid instrument with good internal consistency in assessing patient’s fatigue level among breast cancer and lymphoma patients in Singapore. MFSI-SF’s ability to detect changes in fatigue level following multiple cycles of chemotherapy remains uncertain. However, if needed, total MFSI-SF score rather than individual subscale score should be used to detect changes in patient’s fatigue level. The English and Chinese versions of MFSI-SF also demonstrated comparable equivalence but measurement equivalence was uncertain for the emotional and vigor subscales. Thus, one should be more mindful when interpreting results pooled from these two subscales.

## Additional file

Additional file 1: Supplementary Data. (DOCX 23 kb)

## Abbreviations

CFA: Confirmatory factor analysis
CRF: Cancer-related fatigue
ECOG: An Eastern Cooperative Oncology Group
EFA: Exploratory factor analysis
EORTC-QLQ-C30: The European Organisation for Research and Treatment of Cancer Quality of Life Core Questionnaire 30
ES: Effect size
IRT: Item response theory
MFSI-SF: Multidimensional Fatigue Syndrome Inventory-Short Form
SMR: Standardised response mean
STAI: State-trait anxiety inventory for adults

## Footnotes

1 ECOG Status is a functional scale developed by the Eastern Cooperative Oncology Group in 1982. It serves as a measure of how a disease impacts a patient’s daily living abilities and describes a patient’s level of functioning in terms of their ability to care for themselves, daily activity, and physical ability. 0 corresponds to a patient who is fully active and able to carry out performance without restriction to 5 which represents that the patient is dead [8].
