# Supplementary material for: Psychometric properties and measurement equivalence of the Multidimensional Fatigue Syndrome Inventory- Short Form (MFSI-SF) amongst breast cancer and lymphoma patients in Singapore
Source: Health Qual Life Outcomes. 2018 Jan 19;16:20. doi: 10.1186/s12955-018-0846-6 (PMC5775581; doi:10.1186/s12955-018-0846-6)
Supplement: Additional file 1: — Supplementary Data. (DOCX 23 kb) [file 12955_2018_846_MOESM1_ESM.docx]

| Table S1. Spearman’s correlations between the English and Chinese version of MFSI-SF subscales and EORTC QLQ-C30 domains | | | | | | | | | | | | |
| --- | --- | --- | --- | --- | --- | --- | --- | --- | --- | --- | --- | --- |
| EORTC QLQ-C30 | MFSI-SF | | | | | | | | | | | |
|  | General Fatigue | | Physical Fatigue | | Emotional Fatigue | | Mental Fatigue | | Vigour | | Total Score | |
|  | English | Chinese | English | Chinese | English | Chinese | English | Chinese | English | Chinese | English | Chinese |
| **Functional** |  |  |  |  |  |  |  |  |  |  |  |  |
| Physical | -0.516** | -0.434** | **-0.508**** | **-0.556**** | -0.420** | -0.364** | -0.326** | -0.376** | 0.407** | 0.307** | -0.532** | -0.470** |
| Role | -0.535** | -0.421** | -0.390** | -0.408** | -0.434** | -0.370** | -0.360** | -0.437** | **0.451**** | **0.316**** | -0.525** | -0.438** |
| Emotional | -0.638** | -0.515** | -0.520** | -0.445** | **-0.764**** | **-0.786**** | -0.457** | -0.554** | 0.491** | 0.497** | **-0.664**** | **-0.670**** |
| Cognitive | -0.453** | -0.396** | -0.413** | -0.338** | -0.440** | -0.534** | **-0.790**** | **-0.706**** | 0.320** | 0.330** | -0.524** | -0.514** |
| Social | -0.532** | -0.466** | -0.422** | -0.422** | -0.510** | -0.439** | -0.524** | -0.455** | 0.536** | 0.408** | -0.619** | -0.526** |
| **Global QOL** |  |  |  |  |  |  |  |  |  |  |  |  |
| Global health status (GHS) | -0.569** | -0.523** | -0.525** | -0.545** | -0.501** | -0.469** | -0.421** | -0.463** | 0.633** | 0.621** | -0.669** | **-0.663**** |
| **Symptom scales** |  |  |  |  |  |  |  |  |  |  |  |  |
| Fatigue | **0.634**** | **0.679**** | **0.532**** | **0.547**** | **0.483**** | **0.575**** | **0.390**** | **0.573**** | **-0.438**** | **-0.556**** | **0.588**** | **0.710**** |
| Pain | 0.399** | 0.297** | 0.495** | 0.384** | 0.366** | 0.369** | 0.181* | 0.360** | -0.243** | -0.233* | **0.386**** | **0.366**** |
| **Symptom Items** |  |  |  |  |  |  |  |  |  |  |  |  |
| Dyspnoea | 0.414** | 0.350** | 0.388** | 0.314** | 0.391** | 0.310** | 0.364** | 0.368** | -0.313** | -0.307** | **0.440**** | **0.386**** |
| Insomnia | 0.336** | 0.400** | 0.406** | 0.374** | 0.318** | 0.420** | 0.265** | 0.406** | -0.318** | -0.109 | **0.397**** | **0.362**** |
| Appetite loss | 0.321** | 0.438** | 0.337** | 0.467** | 0.265** | 0.438** | 0.201* | 0.304** | -0.369** | -0.412** | 0.367** | 0.490** |
| Constipation | **0.293**** | **0.444**** | **0.256**** | **0.280**** | **0.202*** | **0.271*** | **0.152** | **0.315**** | **-0.253**** | **-0.321**** | **0.286**** | **0.399**** |
| Nausea & vomiting | 0.228** | 0.237* | 0.221** | 0.238* | 0.182* | 0.286** | 0.078 | 0.212* | -0.219** | -0.300** | 0.249** | 0.328** |
| Diarrhoea | **0.147**** | **0.076** | **0.252**** | **0.245*** | **0.117** | **0.096** | **0.215**** | **0.204** | **-0.125** | **-0.021** | **0.191*** | **0.145** |
| Financial Difficulties | 0.545** | 0.172 | 0.463** | 0.171 | 0.491** | 0.275* | 0.386** | 0.214* | -0.431** | -0.261* | 0.558** | 0.291** |
| MFSI-SF: Multidimensional Fatigue Symptom Inventory- Short Form  EORTC QLQ-C30: European Organization for Research and Treatment of Cancer Quality of Life Core Questionnaire 30  Unmarked correlations were not significant at the 0.05 level  **Correlation was significant at the 0.01 level (2-tailed)  *Correlation was significant at the 0.05 level (2 tailed)  Bolded values indicate hypothesised correlations | | | | | | | | | | | | |

| Table S2. Responsiveness of MFSI-SF total and subscale scores from T1 to T2 | | | | |
| --- | --- | --- | --- | --- |
| MFSI-SF Subscales | Total and subscale MFSI-SF score of English and Chinese version at T1 and T2 (N=224) | | | |
|  | Median score at T1 (IQR) | Median score at T2 (IQR) | Median Change (IQR) | Time in weeks between T1 and T2 (median (IQR)) |
| General | 3.00 (1.00,6.00) | 4.00 (1.00, 7.00) | 0.00 (-1.00, 3.00) | 6.00 (4.00, 6.00) |
| Physical | 2.50 (1.00, 6.00) | 3.00 (1.00, 6.00) | 1.00 (-1.00, 3.00) |  |
| Emotional | 1.00 (0.00, 5.00) | 1.00 (0.00, 5.00) | 0.00 (-1.00, 1.00) |  |
| Mental | 2.00 (0.00, 4.75) | 2.00 (0.00, 5.00) | 0.00 (-1.00, 1.00) |  |
| Vigour | 14.00 (9.00, 19.00) | 14.00 (9.00, 18.00) | 0.00 (-3.00, 3.00) |  |
| Total Score | -3.00 (-15.00, 9.00) | -2.00 (-12.00, 12.00) | 2.00 (-6.00, 10.00) |  |
| MFSI-SF: Multidimensional Fatigue Symptom Inventory- Short Form  None of the median change in scores were clinically significant (median change in scores ≥ 10 points) | | | | |

| Table S3. Comparison of the EORTC QLQ-C30 subdomain scores between the English-speaking and Chinese-speaking patients | | | | |
| --- | --- | --- | --- | --- |
| EORTC QLQ-C30 | Median (IQR) | | | P-value |
|  | Total (N=246) | English-speaking (n=160) | Chinese-speaking (n=86) |  |
| **Global QOL** |  |  |  | 0.122 |
| Global health status | 66.67 (50.00, 83.33) | 66.67 (50.00, 83.33) | 66.67 (50.00, 83.33) |  |
| **Functional** |  |  |  |  |
| Physical | 86.67 (73.33, 93.33) | 80.00 (73.33, 93.33) | 86.67 (73.33, 93.33) | 0.101 |
| Role | 83.33 (66.67, 100.00) | 83.33 (66.67, 100.00) | 83.33 (66.67, 100.00) | 0.268 |
| Emotional | 91.67 (75.00, 100.00) | 91.67 (75.00, 100.00) | 87.50 (75.00, 100.00) | 0.967 |
| Cognitive | 100.00 (83.33, 100,00) | 100.00 (83.33, 100.00) | 83.33 (83.33, 100.00) | 0.676 |
| Social | 83.33 (66.67, 100.00) | 83.33 (66.67, 100.00) | 83.33 (66.67, 100.00) | 0.234 |
| **Symptom scales** |  |  |  |  |
| Fatigue | 33.33 (22.22, 44.44) | 33.33 (22.22, 44.44) | 33.33 (19.44, 44.44) | 0.281 |
| Nausea and vomiting | 0.00 (0.00, 16.67) | 0.00 (0.00, 16.67) | 0.00 (0.00, 4.17) | 0.011^#^ |
| Pain | 16.67 (0.00, 33.33) | 16.67 (0.00, 33.33) | 0.00 (0.00, 33.33) | 0.108 |
| **Single items** |  |  |  |  |
| Dyspnoea | 0.00 (0.00, 33.330 | 0.00 (0.00, 33.33) | 0.00 (0.00, 33.33) | 0.026^#^ |
| Insomnia | 33.33 (0.00, 33.33) | 33.33 (0.00, 33.33) | 33.33 (0.00, 33.33) | 0.282 |
| Appetite | 0.00 (0.00, 33.33) | 0.00 (0.00, 33.33) | 0.00 (0.00, 33.33) | 0.891 |
| Constipation | 0.00 (0.00, 33.33) | 0.00 (0.00, 33.33) | 0.00 (0.00, 33.33) | 0.541 |
| Diarrhoea | 0.00 (0.00, 0.00) | 0.00 (0.00, 0.00) | 0.00 (0.00, 0.00) | 0.771 |
| Financial | 33.33 (0.00, 33.33) | 33.33 (0.00, 33.33) | 33.33 (0.00, 33.33) | 0.331 |
| EORTC QLQ-C30: European Organization for Research and Treatment of Cancer Quality of Life Core Questionnaire 30  ^#^Denotes statically significant different (P< 0.05) through Mann-Whitney U Test | | | | |

| Table S4. Comparison between Mean and Median for total and subscale scores of English and Chinese MFSI-SF versions | | | | | | |
| --- | --- | --- | --- | --- | --- | --- |
|  | Total (N=246) | | English Version (n=160) | | Chinese Version (n=86) | |
|  | Mean ($\pm$ S.D.) | Median (IQR) | Mean ($\pm$ S.D.) | Median (IQR) | Mean ($\pm$ S.D.) | Median (IQR) |
| Total MFSI-SF score | 0.97 (19.04) | -2.00 (-15.00,11.00) | 1.86 (20.41) | -2.00 (-15.00 ,11.00) | -0.67 (16.18) | -3.50 (-15.00,9.25) |
| General | 4.39 (4.56) | 3.00 (1.00,6.00) | 4.96 (4.84) | 4.00 (1.25,6.00) | 3.34 (3.79) | 2.00 (0.00, 5.00) |
| Physical | 3.94 (4.20) | 3.00 (1.00,6.00) | 4.33 (4.65) | 3.00 (1.00,6.00) | 3.23 (3.09) | 2.00 (1.00,5.00) |
| Emotional | 3.20 (4.21) | 1.00 (0.00,5.00) | 3.34 (4.43) | 2.00 (0.00,5.00) | 2.94 (3.76) | 1.00 (0.00,5.00) |
| Mental | 2.97 (3.87) | 2.00 (0.00,5.00) | 3.31 (4.24) | 2.00 (0.00,5.00) | 2.35 (2.97) | 1.00 (0.00,4.00) |
| Vigour | 13.54 (6.52) | 14.00 (8.00,19.00) | 14.08 (6.61) | 14.00 (9.00,19.00) | 12.53 (6.26) | 12.50 (7.00,18.00) |
| MFSI-SF: Multidimensional Fatigue Symptom Inventory- Short Form | | | | | | |
